# Decoding Hydrogen-Affinity-Controlled Surface State Evolution in Sn-Based Single-Atom Alloys for CO_2_ Electroreduction

**DOI:** 10.1021/acs.langmuir.6c04524

**Published:** 2026-10-06

**Authors:** Ruirui Ren, Huitao Fan, Yuhang Wang, Hao Li

**Affiliations:** † College of Chemistry and Pharmaceutical Engineering, 71072 Nanyang Normal University, Nanyang 473061, P. R. China; ‡ Advanced Institute for Materials Research (WPI-AIMR), 13101 Tohoku University, Sendai 980-8577, Japan

## Abstract

The catalytic role of heteroatom dopants in Sn-based electrocatalysts remains elusive because the dynamic evolution of surface states under electrochemical conditions is rarely considered. Herein, density functional theory calculations combined with pH-dependent microkinetic simulations reveal that hydrogen affinity governs the evolution of catalytic surface states in Sn-based single-atom alloys (SAAs) during electrochemical CO_2_ reduction to formate. We demonstrate that hydrogen preferentially occupies either dopant or neighboring Sn sites depending on the electronic structure of the dopant and identify simple electronic descriptors based on the dopant d-band and p-band centers that enable the screening of preferential H adsorption sites. Surface Pourbaix analysis further reveals that Sn-based SAAs tend to evolve into nearly monolayer hydrogen-covered surfaces under practical CO_2_RR conditions, indicating that the catalytic performance should be evaluated on reconstructed rather than pristine surfaces. By incorporating dipole–field interactions, explicit potential of zero charge, and pH-dependent electric-field effects into microkinetic modeling, a predictive pH-dependent activity volcano is established, showing excellent agreement with experimental benchmarking. The resulting framework identifies promising catalysts with distinct pH-dependent activity windows and, more importantly, establishes hydrogen affinity as a fundamental descriptor governing surface-state evolution and thereby influencing catalytic performance, providing a general strategy for the rational design of *p*-block-metal-based electrocatalysts.

## Graphical abstract

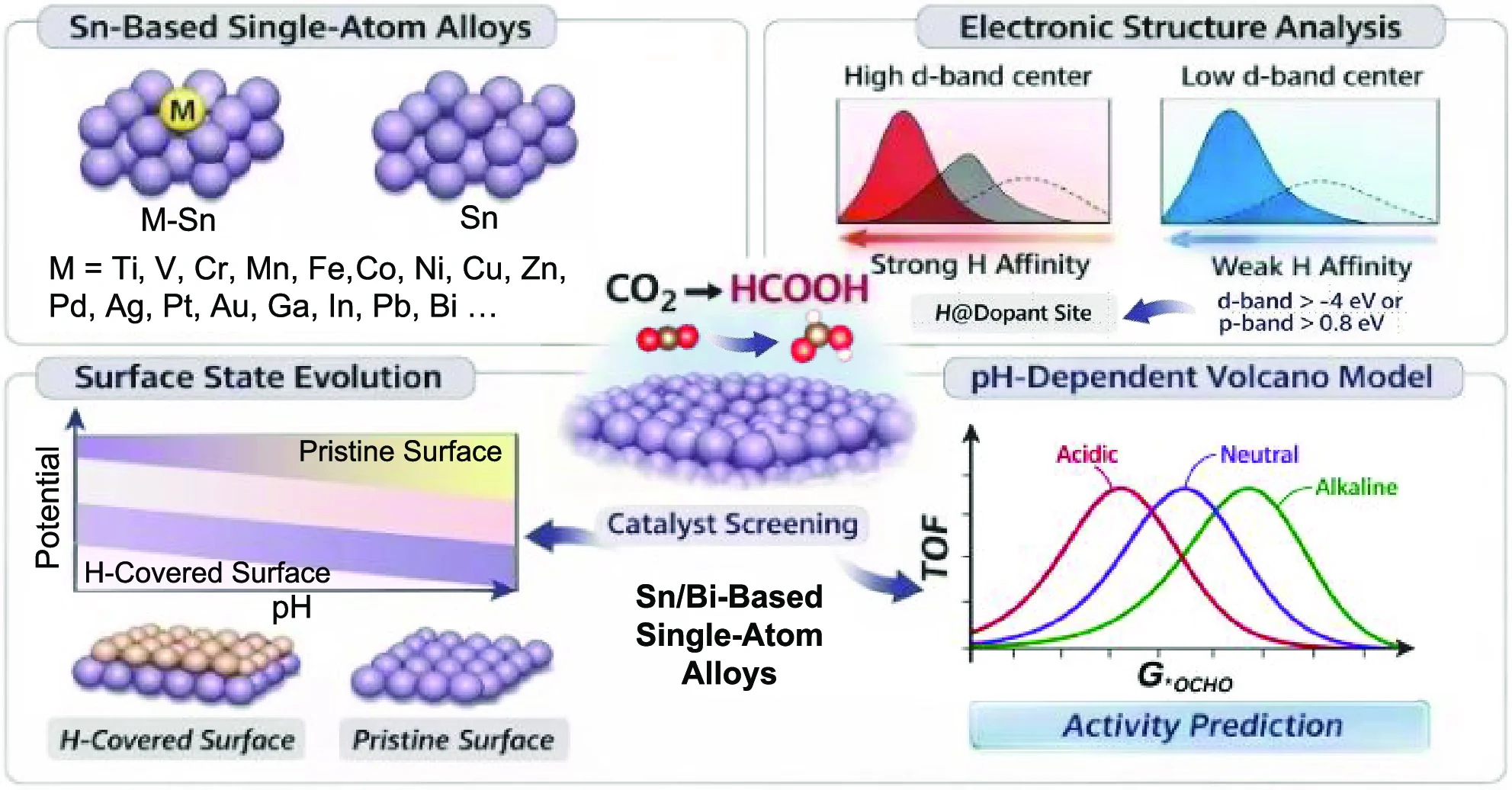

## Introduction

Electrocatalytic CO_2_ reduction (CO_2_RR) can convert CO_2_ into high-value-added chemicals, which is an important pathway to mitigate the greenhouse effect and achieve carbon neutrality.^1–7^ Among various products in the CO_2_RR, the electroreduction of CO_2_ to formic acid (HCOOH) achieves 100% atom utilization. In addition, HCOOH also serves multiple functions, including hydrogen storage, an important chemical feedstock, and fuel, thus attracting widespread attention.^8–10^ Large-scale data mining based on a digital catalysis database (*DigCat*)^11,12^ shows that Sn- and Bi-based catalysts are widely used in CO_2_RR. Statistical analysis of the Faraday efficiency (FE) of the main product shows that most Sn- or Bi-based catalysts exhibit high selectivity for HCOOH (Figure 1a). However, the intrinsic activity of pure metallic Sn or Bi in 0.4 the CO_2_RR-to-HCOOH process is usually limited. Therefore, doping strategies are often used to modify their electronic structures to improve the activity and selectivity of HCOOH formation.^13,14^

**Figure 1:**
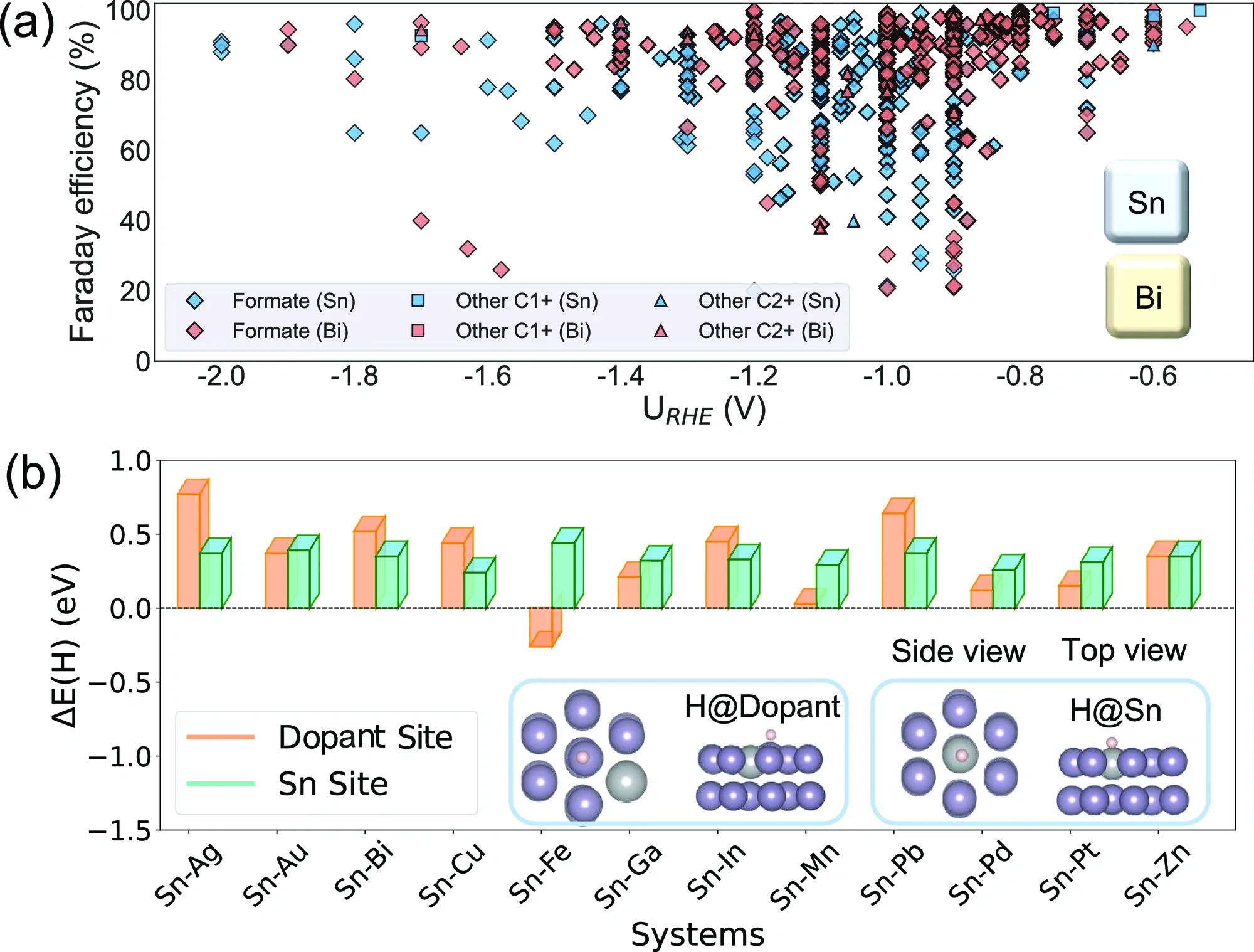
(a) Statistical distribution of the main product Faraday efficiency (FE) of Sn/Bi-based catalysts in CO_2_RR based on large-scale data mining from the *DigCat* database. (b) Comparison of adsorption energies of H at different adsorption sites on the surface of single-atom-doped Sn(100). H@Dopant indicates H adsorption at heteroatom sites; H@Sn indicates H adsorption at Sn sites. Color code: Sn, gray-purple spheres; heteroatom, silver-gray spheres; H, beige spheres.

Numerous experimental and theoretical studies have shown that doping can effectively modulate the activity of the as-designed catalysts.^15^ More recently, orbital engineering and dynamic catalyst reconstruction have emerged as key strategies for regulating the electrocatalytic activity.^16–20^ For example, Chen et al.^21^ synthesized Te-doped Bi nanowires, which achieved high FE for HCOOH production (>90%) at a high current density of 300 mA cm^–2^ in a flow cell reactor. More recently, single-atom alloy (SAA)^22^ strategies have shown remarkable promise. Cai et al.^23^ systematically studied the catalytic performance of Bi-based single-atom alloys (SAAs) in CO_2_RR based on density functional theory (DFT) calculations. By introducing explicit solvation correction, they successfully predicted a series of highly active and selective candidate materials, such as Zn_1_Bi­(012), with a limiting potential as low as −0.14 V. However, under actual electrochemical conditions, changes in the surface coverage often significantly affect catalyst activity.^24,25^ For example, on the Pt(111) surface, H coverage is a critical surface state under the hydrogen evolution reaction (HER) conditions and must be explicitly considered in theoretical modeling.^26^ Similarly, different concentrations of H*-covered states can significantly alter the local electronic structure of Sn and its doped systems, thereby affecting catalytic performance. Nevertheless, systematic studies on the surface coverage effect of doped Sn/Bi-based SAA systems under electrochemical conditions are still lacking.

On the contrary, Sn-based catalysts typically exhibit significant pH-dependent effects. For example, Sun et al.^27^ reported a high-density single-atom Sn catalyst whose CO_2_^–^ adsorption intensity varies with pH, achieving a 90.5% FE for HCOOH and a partial current density as high as 178.5 mA cm^–2^ under acidic conditions. Furthermore, in the Bi_0.1_Sn system, Li et al.^28^ showed that the operating potential shifts positively under alkaline conditions with increasing electrolyte pH. It should be noted that pH changes can modulate the adsorption free energy of reaction intermediates through dipole–field interactions.^29–32^ Therefore, explicitly considering pH-induced free energy changes holds promise for establishing more reliable theory-experimental correlations and achieving more accurate predictions of the catalytic performance.

On the basis of the above understanding, this work takes Sn-based SAAs as representative systems to investigate H-affinity-driven surface-state evolution under electrochemical conditions and its impact on CO_2_RR performance. Specifically, we aim to answer the following three key questions: (1) Under electrochemical conditions, do dopants directly act as reactive sites or mainly play an electronic promoter role? (2) What are the key electronic structure factors that determine the difference in H affinity in the doped Sn system? and (3) How can high-performance Sn/Bi-based SAAs be rationally designed for CO_2_RR? Here, we systematically investigated the CO_2_RR performance of a series of transition- and p-block-metal (Ti, V, Cr, Mn, Fe, Co, Ni, Cu, Zn, Pd, Pt, Ag, Au, Ga, In, Sn, Pb, and Bi)-doped Sn(100) surfaces based on DFT calculations. Strikingly, the results reveal that different dopant atoms exhibit significantly distinct mechanisms in their effects on catalytic performance: some dopants can directly act as reactive sites, while others mainly serve as electronic promoters by regulating the electronic structure of the host Sn. Further electronic structure analysis revealed key descriptors determining the H affinity of the doped system, elucidating the intrinsic electronic origin of the active site selectivity. Most importantly, based on the surface coverage effect, by introducing pH-dependent dipole–field interactions and the potential of zero charge (PZC) at the interface, we constructed a pH-dependent kinetic volcano model. Encouragingly, the theoretical predictions showed good agreement with experimental benchmarking analysis, providing a reliable basis for activity prediction of Sn/Bi-based SAA catalysts. This work not only reveals how electronic-structure-regulated H affinity governs surface-state evolution and modulates the effective catalytic environment but also provides a theoretical foundation for the predictable screening of Sn/Bi-based CO_2_RR catalysts.

## Methods and Computational Details

### DFT Calculation Methods

The Vienna ab initio Simulation Package (VASP) was employed to perform density functional theory (DFT) calculations.^33^ The projector augmented-wave (PAW) method was employed to accurately model ion–electron interactions.^34^ The revised Perdew–Burke–Ernzerhof (RPBE) functional, under the generalized gradient approximation (GGA), was adopted to evaluate exchange–correlation energies.^35,36^ Grimme’s DFT-D3 dispersion correction was used to account for van der Waals interactions between the adsorbates and the surface.^37,38^ The Kohn–Sham wave functions for the valence electrons were expanded using a plane-wave basis set,^39^ with a cutoff energy of 400 eV. The convergence criteria for geometry optimizations were set to a maximum force of 0.02 eV/Å and a total energy change below 10^–5^ eV. A 3 × 3 × 1 grid of k-points was used to sample the first Brillouin zones of the surfaces for structural optimizations. A four-layer metal surface was used, with the bottom two layers fixed to represent the bulk region. Comprehensive information about the computational methods and modeling can be found in the Supporting Information.

### Gibbs Free-Energy Calculations

The adsorption energy of reaction intermediates (ΔE­(*C_*x*_H_*y*_O_*z*_)) during CO_2_ reduction reaction (CO_2_RR) was calculated as^40^

ΔE(Cx*HyOz)=E(Cx*HyOz)−E(*)−x·E(C)−y·E(H)−z·E(O) 1

where *E*(*C_*x*_H_*y*_O_*z*_) and *E*(*) mean the total energy of C_*x*_H_*y*_O_*z*_ species adsorbed on the surface and slab surface (*), respectively. The calculation of *E*(C), *E*(H), and *E*(O) is based on the energies of CO_2_, H_2_, and H_2_O, respectively. The free energy of each elementary step for CO_2_RR was defined by employing previous methods by Nørskov and his collaborators:^41^

2 $$\Delta G=\Delta E+\Delta E_{\mathrm{ZPE}}+\int C_{p}dT-T\cdot \Delta S+E_{\mathrm{solv}}$$

where Δ*E* means the difference in DFT electronic energy, Δ*E*_ZPE_, ∫*C*_p_d*T*, and Δ*S* refer to the zero-point energy, heat capacities, and entropy corrections, and *T* is the absolute temperature, set to 298.15 K. The zero-point energy and entropic contribution (*T*Δ*S*) were obtained with the harmonic approximation. *E*_solv_ is the solvation corrections described in ref 42, with Δ*E*_solv_(OCHO) = −0.1 eV, Δ*E*_solv_(COOH) = −0.25 eV, and Δ*E*_solv_(CO) = −0.1 eV. To improve the energetics of gas-phase species, energy corrections of 0.29 eV for CO_2_ and 0.09 eV for H_2_ were included to account for systematic DFT errors.^43^ The DFT-calculated energies, zero-point energies, heat capacities, and entropic contributions for CO_2_RR are taken from our previous work (as listed in Table S5).^40^

### Surface Pourbaix Diagram Calculations

The free energies *G*_SP_ presented in the surface Pourbaix diagrams were computed using the approach outlined in refs 44–46

3 $$G_{\mathrm{SP}}=G_{\mathrm{bare}}+mG_{H_{2}O}-G_{\mathrm{tot}}-\left(2m-n\right)\left(\frac{1}{2}G_{H_{2}}-U_{\mathrm{SHE}}-2.303k_{B}T^{\;}\times \mathrm{pH}\right)$$

where G_bare_ is the total free energy of a bare surface, G_H_2_O_ is the free energy of H_2_O, G_tot_ is the total free energy of the surface with adsorbate/nonmetal vacancies, and G_H_2__ is the total energy of H_2_. *m* and *n* represent the numbers of oxygen and hydrogen atoms adsorbed on a stoichiometric surface, respectively, and *k*_B_ is the Boltzmann constant.

### Simulating pH with Electric Field

Under each applied electric field, adsorbates were relaxed until the force convergence criterion of 0.02 eV/Å was achieved. The lowest-energy structure was then used to calculate the adsorption energy at that field. To account for the effects of potential and pH, we linked the electric fields to SHE (SHE: Standard Hydrogen Electrode) and RHE (RHE: Reversible Hydrogen Electrode) potentials using a parallel-plate capacitor model. As described in eq 4, this model defines σ as the charge density, ε_0_ as the vacuum permittivity (8.85 × 10^–12^ F m^–1^), ε as the dielectric constant (unitless), *C*_H_ as the Helmholtz capacitance (μF cm^–2^), *U*_SHE_ as the potential relative to the SHE, and *U*_PZC_ as the potential at the PZC at the SHE scale. Fumagalli et al.^47^ demonstrated that the dielectric constant of water near a surface is two. The Helmholtz capacitance (*C*_H_) can vary with the surface and potential, and was determined to be 25 μF cm^–2^ as described in refs 31,48,49,31,48,49

4 $$\mathrm{E⃗}=\frac{\sigma }{\varepsilon \varepsilon_{0}}=\frac{C_{H}\left(U_{\mathrm{SHE}}-U_{\mathrm{PZC}}\right)}{\varepsilon \varepsilon_{0}}$$

To assess the response of the adsorption energy to the electric field, a second-order polynomial was fitted to the calculations for each adsorbate over the field range. Equation 5 was then utilized to determine the intrinsic dipole moment (μ) and polarizability (α):

5 $$G_{\mathrm{ads}}=G_{\mathrm{ads}}^{\mathrm{PZC}}+\mu \mathrm{E⃗}-\frac{\alpha }{2}{\mathrm{E⃗}}^{2}$$

where G_PZC_ represents the binding free energy of the adsorbate at the PZC, corresponding to the energy calculated without an applied electric field.

Since the electrode potential can be referenced to either SHE or RHE, the two scales are related by eq 6:

6 $$U_{\mathrm{SHE}}=U_{\mathrm{RHE}}-k_{B}T\;\ln\left(10\right)\times \mathrm{pH}=U_{\mathrm{RHE}}-0.059\mathrm{pH}$$

Ultimately, the free energy of adsorbate at the given *U*_RHE_ and *U*_SHE_ is shown by eq 7:^50^

7 $$G_{\mathrm{ads}}=G_{\mathrm{ads}}^{\mathrm{PZC}}+\mu \frac{C_{H}\left(U_{\mathrm{RHE}}-0.059\mathrm{pH}-U_{\mathrm{PZC}}\right)}{\varepsilon \varepsilon_{0}}-\frac{\alpha }{2}{\left(\frac{C_{H}\left(U_{\mathrm{RHE}}-0.059\mathrm{pH}-U_{\mathrm{PZC}}\right)}{\varepsilon \varepsilon_{0}}\right)}^{2}$$

### pH-Dependent Activity Modeling

The microkinetic modeling of the CO_2_RR volcano was based on the approach outlined by Hansen et al.^51^ and Kelly et al.^48^ Rates for intermediate steps were calculated as below:

8 $$\mathrm{rate}=k_{f}\prod \theta_{\mathrm{reac}}-k_{b}\prod \theta_{\mathrm{prod}}$$

where θ_reac_ and θ_prod_ are the coverages of reactants and products, respectively. *k*_f_ and *k*_b_ are the rate constants of the forward and backward reaction, which were calculated as the function of pre-exponential factor *A* (s^–1^), activation free energy G_a_, Boltzmann constant *k*_B_, and reaction temperature *T*:

9 $$k=Ae^{-G_{a}/k_{B}T}$$

The intermediates for HCOOH formation considered in the modeling are as follows:

$${\mathrm{CO}}_{2}\left(g\right)+*+H^{+}+e^{-}\to *\mathrm{OCHO}$$ 10

$$*\mathrm{OCHO}+H^{+}+e^{-}\to *+\mathrm{HCOOH}\left(l\right)$$ 11

Reactions 10 and 11 involve proton–electron transfer steps, where the energy of the proton–electron pair is represented by the energy of half of a H_2_ molecule according to the CHE method.^41^ Besides, we incorporate an intrinsic barrier of 0.26 eV to the free-energy diagram to correlate the thermodynamic to the kinetic, which is commonly used in electrocatalysis studies to account for the proton–electron transfer step.^30,31,51^ An additional free energy barrier of 0.54 eV was set to account for solvent reorganization.^40^

## Results and Discussion

### H Affinity Analysis of Sn-Based SAAs

Under CO_2_RR conditions, pure metal or alloy surfaces typically tend to be covered by H, but whether H occupation preferentially occurs at doped sites or Sn sites remains to be clarified. Therefore, we first assessed the affinity of H by comparing the adsorption energies of H (Δ*E*(H)) at different sites. During geometry optimization, six Sn-based SAAs underwent significant structural deformation and were therefore excluded from subsequent analysis. The adsorption energies of H at different sites in the remaining systems are compared in Figure 1b. The corresponding adsorption configurations are summarized in Figures S1 and S2. The results show that in systems like Sn–Au/Sn-Fe, Δ*E*(H) is lower at doped sites than at Sn sites (a more negative Δ*E*(H) indicates a stronger H affinity), indicating that under electrochemical conditions, doped sites are more likely to be preferentially occupied by H. This result supports our hypothesis that in some SAA systems, dopant atoms may not directly act as reactive centers but are more likely to function as electronic modifiers, and therefore need to be carefully considered in catalytic mechanism analysis.

To elucidate the origin of different H affinities, we conducted a systematic electronic structure analysis of Sn-based SAAs. The results show that the band center of the Sn sites varies little in various doped systems (Figure 2), while the preference for H adsorption sites is closely related to the band center position of the dopant element: when the dopant element satisfies the condition that the d-band center is higher than −4 eV (Figure 2a–d) or the p-band center is higher than 0.8 eV (as summarized in Table S1), the system as a whole tends to form a configuration where H preferentially occupies heteroatom sites; conversely, H is more likely to preferentially adsorb at the Sn site. This rule shows good applicability in various doped systems (such as Ag, Cu, In, Zn, Pt, Pd, Fe, etc.), indicating that the band center of the doped atom can serve as an effective electronic structure descriptor for describing the preference of H adsorption sites.

**Figure 2:**
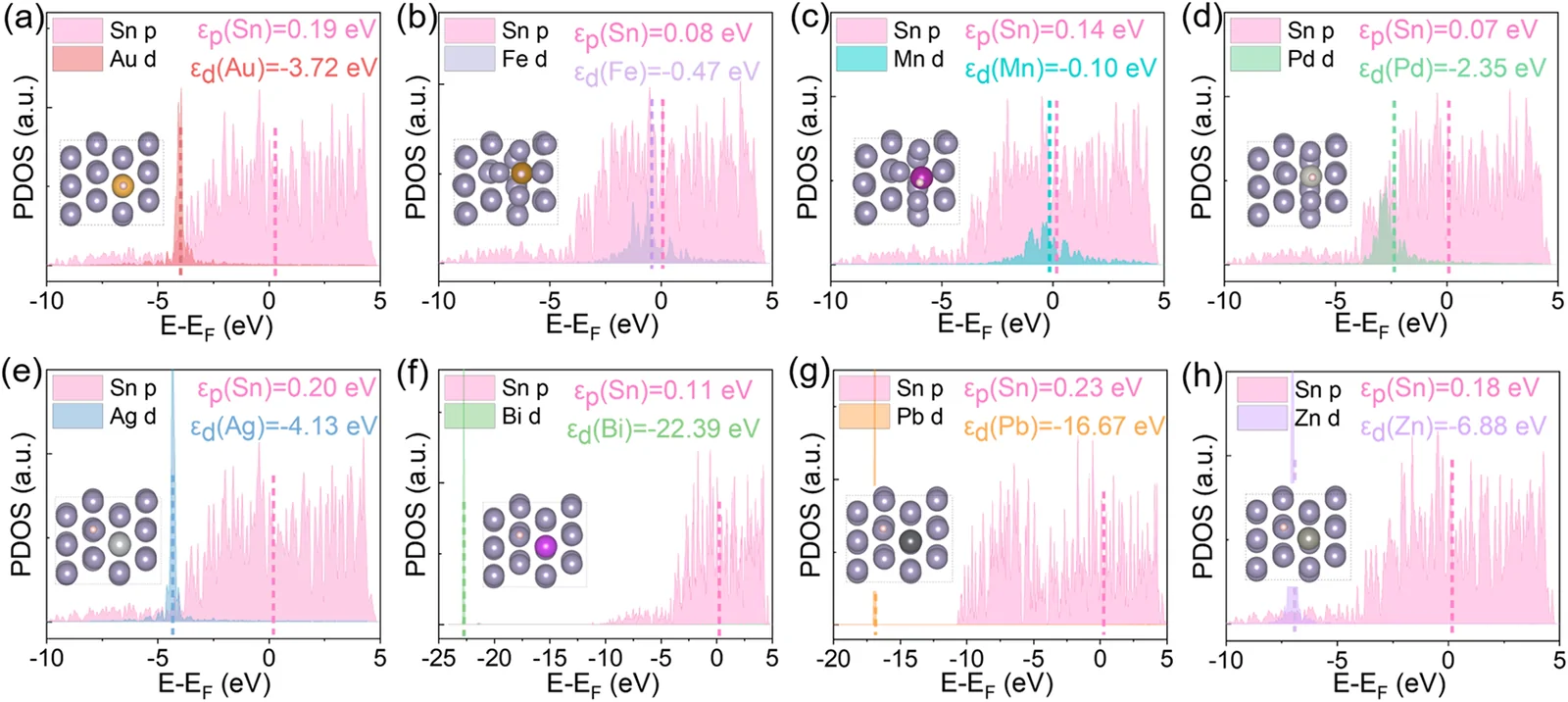
Partial density of states (PDOS) of representative doped Sn systems: (a) Sn–Au, (b) Sn–Fe, (c) Sn–Mn, (d) Sn–Pd, (e) Sn–Ag, (f) Sn–Bi, (g) Sn–Pb, and (h) Sn–Zn. In panels (a–d), H preferentially adsorbs at the heteroatom sites, whereas in panels (e–h), H preferentially adsorbs at the Sn sites. Insets show the top views of the most stable H adsorption configurations. Color scheme: Sn, grayish-purple; H, off-white; heteroatoms, element-specific colors.

Theoretically, this pattern stems from the difference in hybridization strength between the dopant orbitals and the H 1s orbitals. When a dopant element has a high d-band center (greater than −4 eV), its d-states have a high density of states near the Fermi level, making it easier for them to effectively overlap with the H 1s orbitals, thus forming stable M–H bonds and allowing H to preferentially occupy heteroatom sites. For example, in the Sn–Au and Sn–Fe systems, the d-band centers of Au (−3.72 eV) and Fe (−0.47 eV) are both higher than −4 eV, and both exhibit H preferentially occupying heteroatom sites (Figure 2a–d). Conversely, for dopant elements with d-band centers much lower than −4 eV (such as Sn–Ag, Sn–Bi, Sn–Pb, and Sn–Zn), their d-state energy levels are deeper and usually exhibit full-shell characteristics, making it difficult for them to effectively participate in bonding. Therefore, H tends to adsorb at Sn sites (Figure 2e–h).

On the other hand, for *p*-block-doped elements, the preference for H adsorption sites is also closely related to the p-band center. When the p-band center is high (e.g., Ga, p-band center = 0.84 eV, Table S1), the dopant atom has a higher density of states near the Fermi level that participates in bonding, thereby enhancing the overlap between H 1s and p orbitals and making H more likely to adsorb directly onto heteroatom sites. Conversely, when the p-band center is low (e.g., <0.8 eV), heteroatoms tend to induce local charge redistribution through p–p interactions, thereby modulating the electronic structure of neighboring Sn atoms. In this case, the dopant atom usually does not directly act as the most stable adsorption site but rather changes the H adsorption strength of Sn sites through electronic modulation effects, exhibiting a typical electronic promoter role. For example, in the Sn–In system, the p-band center of In is 0.75 eV, and H preferentially occupies Sn sites.

Therefore, in Sn-based SAAs, dopants do not always directly act as adsorption centers but may indirectly affect the stability of reaction intermediates by modulating the local electronic structure of the host surface. On the basis of electronic structure analysis, we propose using d-band centers of >−4 eV (Figures 2a–d and S9) as the primary criterion, combined with p-band centers above 0.8 eV (Table S1) as an auxiliary criterion, to uniformly describe the preference of H adsorption sites in Sn-based single-atom alloys. This electronic structure descriptor provides a quantifiable predictive capability for distinguishing the true active centers (doped atoms or host Sn) in doped Sn-based systems.

### Surface State Analysis

Determining the actual surface states of Sn-based SAA catalysts under electrochemical conditions is a prerequisite for establishing structure–performance relationships. Considering the negative potentials relevant to the CO_2_RR, *O and *OH species, which are generally favored at more positive potentials, were not included in the surface Pourbaix analysis. Therefore, based on the differences in H affinity, we further analyzed the evolution of surface H coverage in Sn-based SAAs. Figures 3a–f and S3–S8 show the two-dimensional and one-dimensional surface Pourbaix diagrams of six representative Sn-based SAAs. Tables S2 and S3 summarize the surface coverage statistics of Sn-based SAAs systems across different potential ranges. Figure 3a–c represents systems where H preferentially occupies Sn sites, and Figure 3d–f represents systems where H preferentially occupies heteroatom sites. The results show that as the applied potential gradually shifts negatively, the Sn SAA surface gradually transforms from a pristine structure to a highly H-covered structure, and the surface H coverage increases continuously with decreasing potential. Especially under conditions of approximately −1 V/SHE (standard hydrogen electrode, typical CO_2_RR working potential),^40^ the surface configuration containing 11–12 *H (adsorbed H) is thermodynamically more stable. Taking Sn–Ga as an example, when the potential is reduced from 2 V to −2 V/SHE, the system eventually forms a fully H-covered surface (1 ML H coverage) (Figure 3g). Therefore, at the operating potential, we can conclude that Sn-based SAAs tend to form a monolayer of H-covered structure, indicating that traditional structure–performance analysis based on pristine surfaces is difficult to accurately describe the real reaction environment.^52^ In this context, H affinity primarily serves as a surface-state descriptor rather than a direct activity descriptor, as it determines the preferential H occupation and thereby the electrochemically relevant catalytic environment. On the basis of surface state analysis, all structure–activity relationship analyses below are based on the electrochemically reconstructed H-covered surface model, thereby achieving a more physically meaningful catalytic performance evaluation.

**Figure 3:**
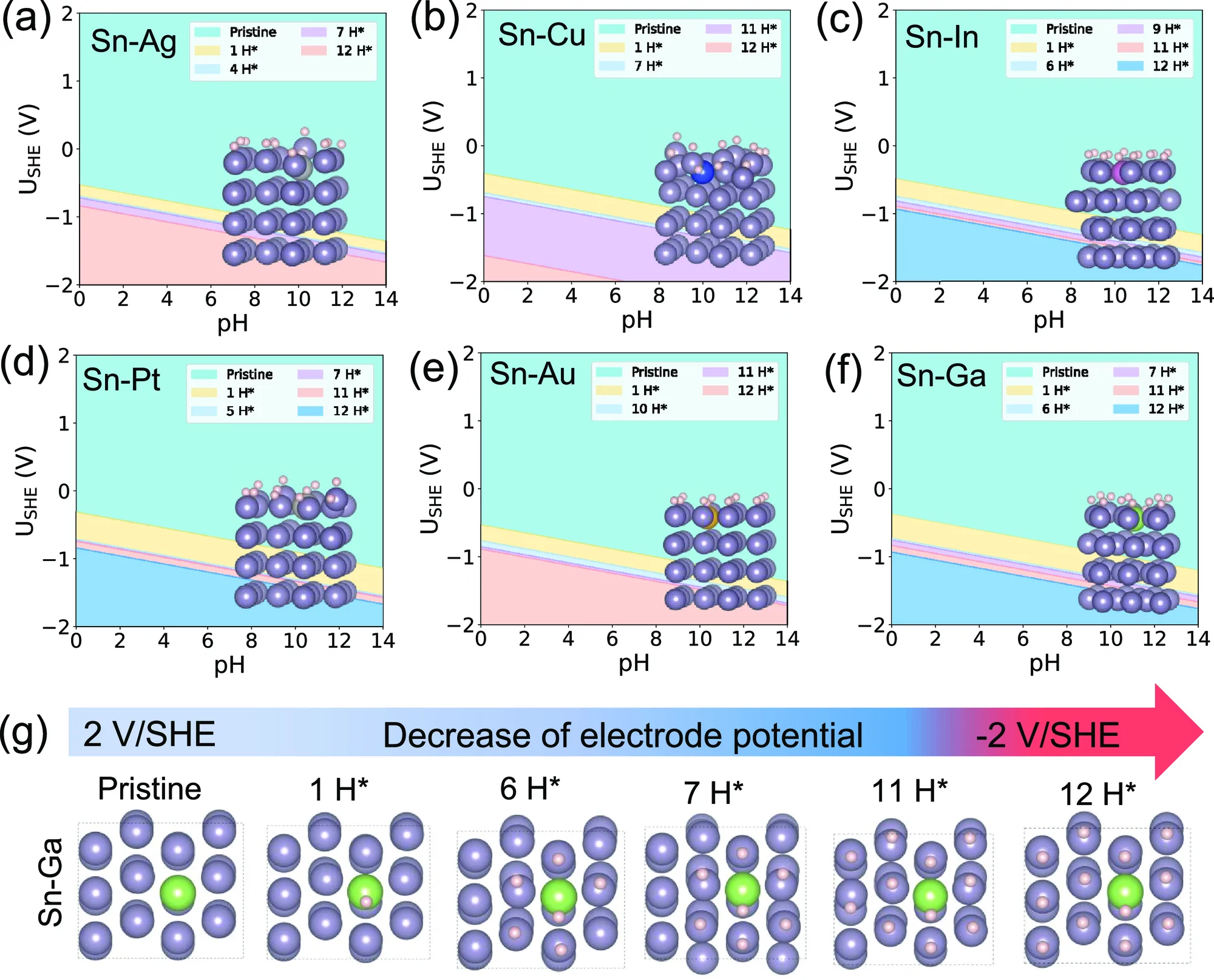
Two-dimensional surface Pourbaix diagrams of doped systems where H preferentially occupies Sn sites: (a) Sn–Ag; (b) Sn–Cu; and (c) Sn–In. Two-dimensional surface diagrams of doped systems where H preferentially occupies heteroatom sites: (d) Sn–Pt; (e) Sn–Au; (f) Sn–Ga. (g) Schematic diagram of the surface covering structure of the Sn–Ga system as a function of potential from 2 V/SHE → −2 V/SHE. Pristine denotes the H-free surface, whereas n H* denotes a surface state containing n adsorbed H atoms. All structures are top views. Color scheme: Sn is represented by grayish-purple spheres, H by beige spheres, Cu by blue spheres, In by pink spheres, Pt by gray spheres, Au by orange spheres, and Ga by green spheres.

### pH-Dependent Microkinetic Modeling

Thermodynamic calculations based on the traditional computational hydrogen electrode (CHE) model struggle to accurately describe pH-dependent free energy changes; thus, a 0.059 × pH term is typically introduced to correct the reaction free energy.^53,54^ However, even under fixed U_RHE_ conditions, pH variations may cause significant shifts in the activity volcano, affecting the reliable prediction of activity trends.^55^ To more accurately describe electrochemical interfacial effects, we systematically investigated the reaction mechanism of Sn-based SAAs under CO_2_RR conditions by combining pH-dependent microkinetic analysis.

The presence of electric fields can significantly alter the adsorption strength of reaction intermediates through dipole–field interactions.^56,57^ Therefore, determining the response of *OCHO to an electric field is crucial for understanding pH-dependent kinetics. Figure 4a–f illustrates the response of *OCHO adsorption free energy to an applied electric field in representative systems. The vertical axis represents the change in free energy relative to the absence of an electric field (e.g., ΔG_ads_ at PZC): positive values indicate weakened *OCHO adsorption under an applied electric field, while negative values indicate that the electric field stabilizes the *OCHO intermediate. The quadratic fitting curves show that all six systems exhibit a significant stabilizing effect under more negative electric fields due to the fitted dipole moment and polarizability of adsorbed *OCHO. Furthermore, it should be noted that more negative electric fields typically correspond to more alkaline reaction environments. Thus, in another word, as the pH increases, the adsorption free energy of *OCHO tends to be more negative. This result indicates that electric-field-induced changes in the adsorption energies of reaction intermediates can potentially alter the rate-determining step. To establish a unified volcano model, the averaged values of the dipole moments and polarizabilities across different systems were employed in the subsequent derivation of the theoretical volcano relationship.

**Figure 4:**
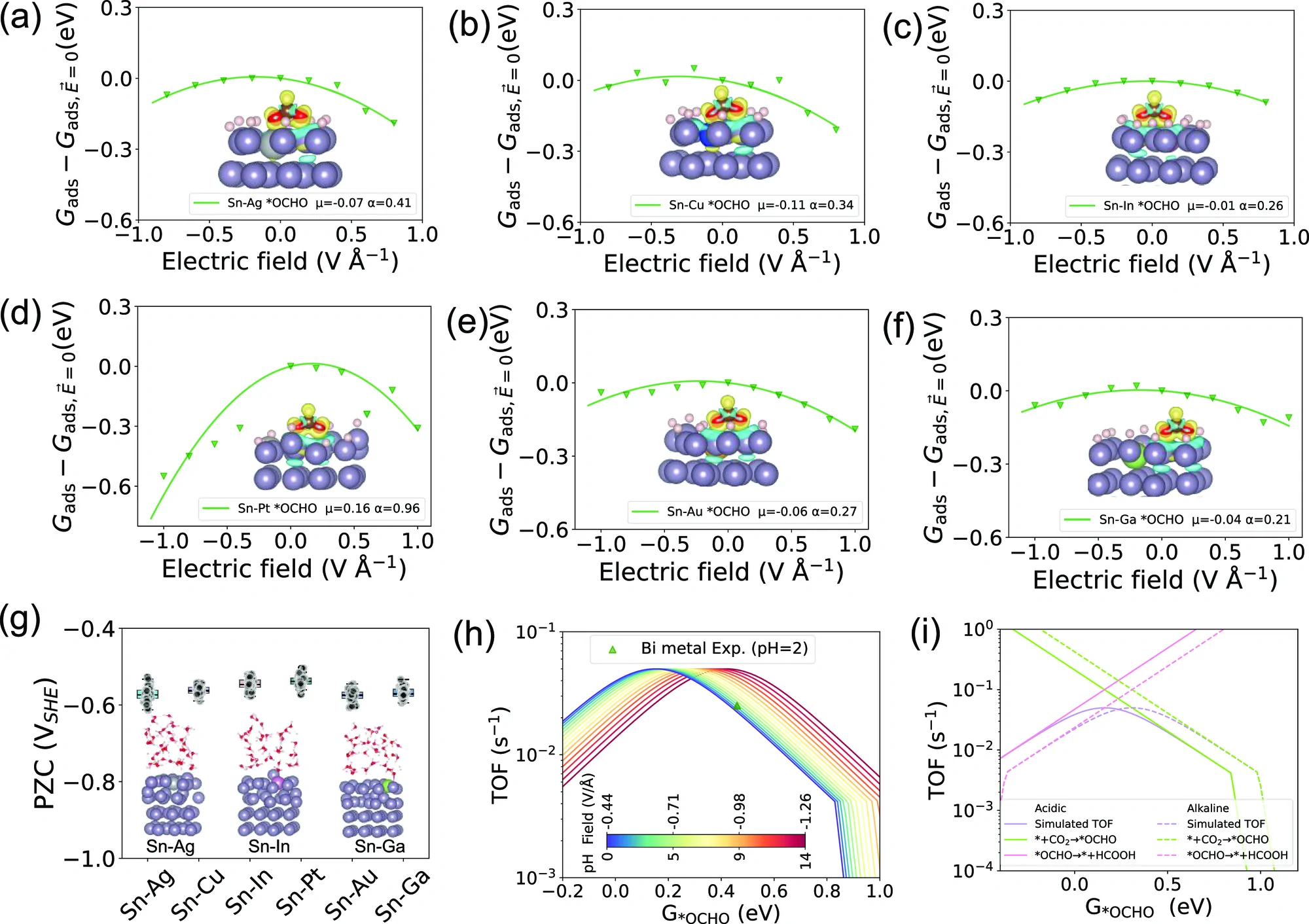
Effect of electric field on the adsorption free energy of OCHO (G_*OCHO_) and its quadratic function fitting results: (a–c) Systems where Sn sites are preferentially occupied. (d–f) Systems where heteroatom sites are preferentially covered. μ is the dipole moment (e Å) and α is the polarizability (e^2^ V^–1^). (g) Potential of zero-charge (PZC) calculated based on explicit solvent models. The inset shows the explicit solvent models for Sn–Ag, Sn–In, and Sn–Ga. (h) pH-dependent microkinetic simulation of CO_2_RR-to-HCOOH. Lower electric fields correspond to higher pH, and higher electric fields correspond to lower pH. (i) Rate-determining step analysis of CO_2_RR to HCOOH under acidic (dashed line) and alkaline (solid line) conditions. For the purple curve, the rate-limiting step is * + CO_2_ → *OCHO, whereas for the green curve, it is *OCHO → * + HCOOH.

Potential of zero charge (PZC) is a key parameter determining the electrochemical behavior of the metal–water interface.^32,49,59^ It directly affects the interfacial electric field strength at a given potential and further modulates the electric double-layer structure.^31^ Based on the parallel-plate capacitance model, a quantitative relationship between the electric field strength and the electrode potential (vs SHE) can be established.^48^ Figure 4g shows the PZC distribution results obtained by *ab initio* molecular dynamics (AIMD) simulation. Despite the introduction of different dopants, the overall PZC of Sn-based SAAs shows small variations, ranging from −0.54 to −0.57 V. These minor differences mainly stem from variations in metal–water interactions in different systems; for example, the formation of some metal–O configurations can be observed on the Sn–Ga and Sn–Pt (as shown in Figure S10) surfaces. This result is consistent with other reported systems, such as the average PZC of reconstructed SnS surfaces, which is approximately −0.61 V, and the presence of certain metal–O interfacial interactions,^40^ indicating that Sn-based systems share certain commonalities in electrochemical interfacial properties.

Considering these factors, we constructed the pH-dependent model for Sn/Bi-based SAA catalysts using the kinetic and thermodynamic parameters of each elementary reaction step in CO_2_RR. To establish a unified description, we used the average PZC value calculated by the AIMD as the input parameter. The resulting kinetic volcano plot is shown in Figure 4h. Compared with traditional thermodynamic-only models, the volcano curve shows a significant rightward shift when transitioning from low-pH to high-pH conditions. This is mainly due to the further stabilization effect of *OCHO under more negative interfacial electric fields, which alters the scaling relationship between the reaction intermediates and causes a systematic shift in the volcano peak position. In particular, the volcano peak position shifts by approximately 0.30 eV when the pH changes from 0 to 14. More importantly, the theoretical predictions and experimentally measured turnover frequencies (TOFs) show good agreement,^60^ indicating that the established model can accurately describe the catalytic performance of Sn/Bi-based SAAs under different pH conditions (Figure 4h).

Figure 4i illustrates the rate-determining step (RDS) analysis of the CO_2_RR-to-HCOOH process. When G_*OCHO_ < 0.1 eV, the RDS is *OCHO → HCOOH due to the strong adsorption of *OCHO and the further enhancement by the dipole–field stabilization effect; the reaction activity decreases with increasing pH. When G_*OCHO_ > 0.4 eV, the * + CO_2_ → *OCHO becomes the RDS due to the positive adsorption free energy of *OCHO; within this range, increasing pH lowers the free-energy requirement for *OCHO formation, thereby enhancing the reaction activity. In the intermediate region of ∼0.1–0.4 eV, the system exhibits superior overall catalytic activity due to the change in RDS. Overall, the RDS analysis provides clear guidance for the rational design of Sn/Bi-based SAAs catalysts.

### Prediction

Bi­(012) surfaces have been widely investigated in both theoretical and experimental CO_2_RR research.^61^ To examine whether high H coverage also occurs in other CO_2_RR systems, we further evaluated the surface state of Bi(012). (Figures 5a and S11). The results show that forming a 1 ML H-covered structure is thermodynamically favorable under electrochemical CO_2_RR conditions, indicating the necessity of explicitly considering the H-covered effect before the performance evaluation. Furthermore, to achieve efficient catalyst design, we constructed and analyzed 29 Sn/Bi-based SAAs systems. Figure 5b,c show the selectivity relationships between G_*OCHO_ and G_*COOH_, and between G_*OCHO_ and G_*H_. The results indicate that the predicted systems as a whole exhibit excellent CO_2_RR-to-HCOOH selectivity; detailed data are shown in Table S4. Encouragingly, most of the designed Sn/Bi SAAs showed a significant preference for *OCHO, indicating that HCOOH formation is thermodynamically more favorable. More importantly, based on the constructed pH-dependent volcano model, we screened several candidate catalysts that performed well under different pH conditions in the CO_2_RR reaction (−1 V/RHE) (Figure 5d). Representative single-atom Sn/Bi-based SAAs with adsorbed OCHO are shown in Figure 5e. For example, Pb@Sn(100) (G_*OCHO_ = 0.46 eV) showed high activity at pH = 14; V@Bi(012) (G_*OCHO_ = 0.26 eV) exhibited good performance over a wide pH range; and Zn@Bi(012) (G_*OCHO_ = 0.16 eV) showed outstanding performance at pH = 1. These distinct optimal pH ranges originate mainly from their different *OCHO adsorption strengths. As the pH increases, the pH-dependent interfacial electric field effect further stabilizes *OCHO, shifting the volcano optimum toward weaker intrinsic *OCHO adsorption; consequently, Zn@Bi(012) is favored under acidic conditions, whereas Pb@Sn(100) becomes more favorable under alkaline conditions, with V@Bi(012) exhibiting intermediate behavior. Overall, this study provides theoretical guidance for the rational design and precise screening of Sn- and Bi-based catalysts by explicitly introducing the pH effect.

**Figure 5:**
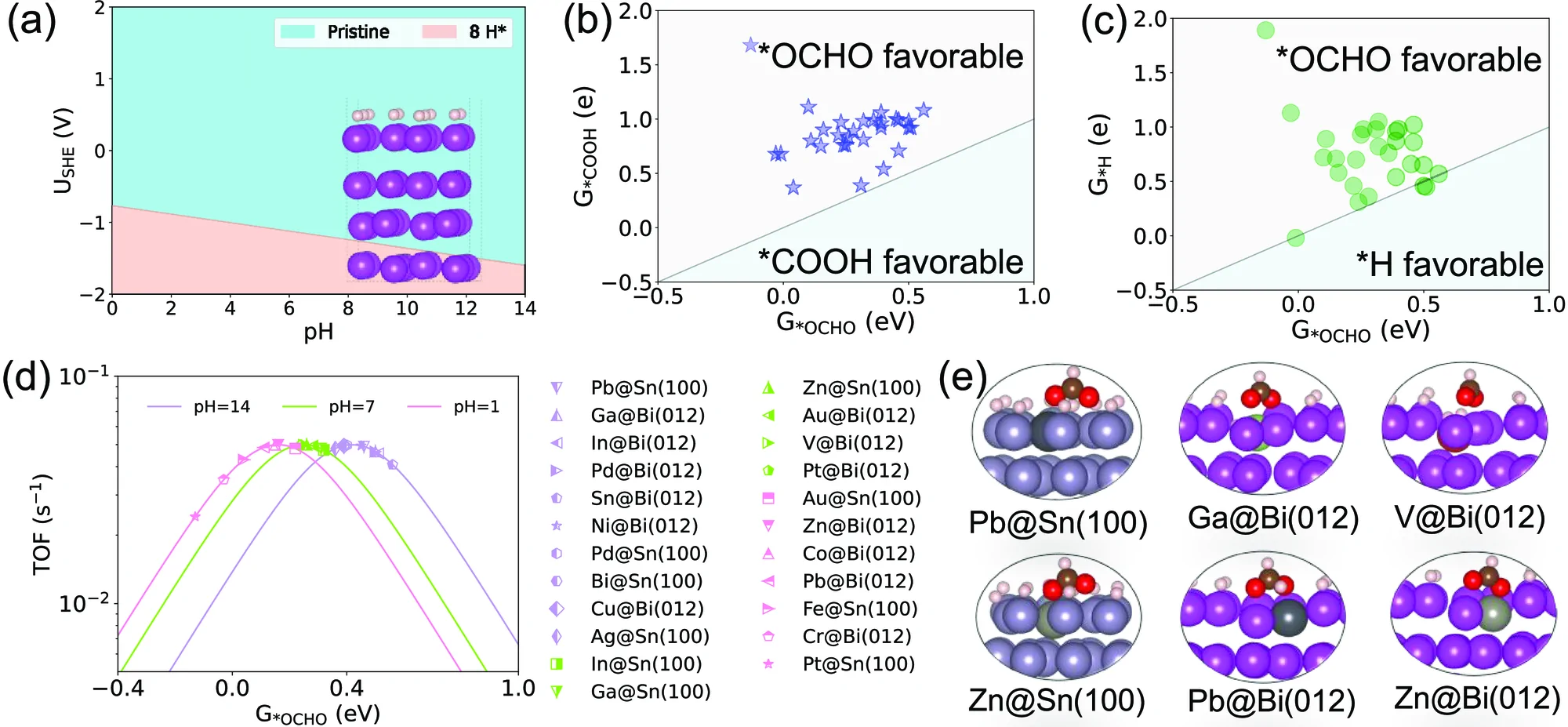
(a) Two-dimensional Pourbaix diagram of the Bi(012) surface. (b, c) Selectivity analysis of doped Sn/Bi-based SAAs catalysts: comparison of G_*OCHO_ and G_*COOH_, and G_*OCHO_ and G_*H_. G_*OCHO_, G_*COOH_, and G_*H_ denote the adsorption free energies of *OCHO, *COOH, and *H, respectively. (d) pH-dependent volcano plot of HCOOH formation at −1.0 V/RHE under different pH conditions. (e) Schematic illustration of the optimized *OCHO adsorption configurations on representative single-atom Sn/Bi-based SAAs. Color code: Bi is a purple sphere, Pb is a brown sphere, V is a dark red sphere, Zn is a dark gray sphere, and O is a bright red sphere.

## Conclusions

In summary, this work reveals that hydrogen affinity serves as a fundamental factor governing the evolution of catalytic surface states in Sn-based single-atom alloys under electrochemical conditions. Rather than acting universally as catalytically active sites, heteroatom dopants regulate catalytic behavior through distinct hydrogen-affinity-dependent mechanisms, leading to different hydrogen occupation preferences and active-site evolution pathways. We started with the investigation of heterometallic Sn-based SAAs using DFT calculations, revealing key electronic structure features that regulate H affinity: when the d-band center of the dopant element is higher than −4 eV or the p-band center is higher than 0.8 eV, the heteroatom exhibits stronger H affinity, thereby inducing a H-affinity-dominated active site transition, i.e., H preferentially occupies heteroatom sites and regulates the catalytic behavior of neighboring Sn sites. Furthermore, by constructing a pH-electric field-coupled microkinetic model, we established a pH-dependent kinetic volcano plot, which showed good consistency with the experimental benchmarking results in TOF. On the basis of this theoretical framework, we screened a series of Sn/Bi-based SAA catalysts with excellent performance under different pH conditions. For example, under a CO_2_RR potential of −1 V/RHE, Pb@Sn(100) (G_*OCHO_ = 0.46 eV) exhibits high activity at pH = 14, whereas Zn@Bi(012) (G_*OCHO_ = 0.16 eV) shows outstanding performance under acidic conditions (pH = 1). Overall, this study shows that the true function of dopants needs to be systematically evaluated in conjunction with their H affinity and the resulting surface coverage evolution under electrochemical CO_2_RR conditions; the proposed pH-dependent microkinetic model provides predictive theoretical guidance for the rational design of high-performance Sn/Bi-SAAs catalysts in the CO_2_RR-to-HCOOH.

## Supplementary Material



## Notes

Additional computational details; optimized H adsorption configurations; surface Pourbaix diagrams and coverage statistics; PDOS and band-center data; AIMD-sampled metal–water interfacial structures; H-affinity data; intermediate Gibbs free energies; and thermodynamic corrections. (PDF)
