# Supplementary material for: Decoding Hydrogen-Affinity-Controlled Surface State Evolution in Sn-Based Single-Atom Alloys for CO2 Electroreduction
Source: Langmuir. 2026 Oct 6;42(41):30954–63. doi: 10.1021/acs.langmuir.6c04524 (PMC13689927; doi:10.1021/acs.langmuir.6c04524)
Supplement: Supplementary file 1 [file la6c04524_si_001.pdf]

Supplementary Information

Decoding Hydrogen-Affinity-Controlled Surface State Evolution in Sn-Based Single-Atom Alloys for CO_2 Electroreduction

Ruirui Ren,[a] Huitao Fan,[a,*] Yuhang Wang,[a,b,*] Hao Li,[b,*]

[a] *College of Chemistry and Pharmaceutical Engineering, Nanyang Normal University, Nanyang 473061, PR China*

[b] *Advanced Institute for Materials Research (WPI-AIMR), Tohoku University, Sendai, 980-8577, Japan*

* Corresponding Authors:

Email: fanhuitao818@nynu.edu.cn (H. F.)

Email: yhwang22-c@my.cityu.edu.hk (Y. W.)

Email: li.hao.b8@tohoku.ac.jp (H. L.)

Supplementary Information

1. Computational details 3

1.1 Charge Density Difference Calculation 3

1.2 Calculation of the Potential of Zero Charge 3

2. Supplementary tables and figures 4

Figure S1 4

Figure S2 5

Figure S3 6

Figure S4 7

Figure S5 8

Figure S6 9

Figure S7 10

Figure S8 11

Figure S9 12

Figure S10 13

Figure S11 14

Table S1 15

Table S2 16

Table S3 17

Table S4 18

Table S5 20

References 21

1. Computational details

1.1 Charge Density Difference Calculation

The charge density differences (Δρ) were calculated using the following equation:

$$\Delta\rho = \rho_{\mathrm{tot}} - \rho_{\mathrm{bare}} - \rho_{OCHO}, (1)$$

where ρ_{tot} is the charge density of the surface with the adsorbed OCHO, ρ_{bare} is the charge density of the bare surface, and ρ_{OCHO} is the charge density of the OCHO molecule.

1.2 Calculation of the Potential of Zero Charge

AIMD simulations of metal-water interfaces have emerged as the more accurate method to estimate PZCs.[1-4] AIMD simulations were performed with VASP using the RPBE exchange-correlation functional,[5, 6] and included the D3 dispersion correction scheme,[7] recognized for its precision in describing metal-water interfaces.[8, 9] A plane-wave cutoff energy of 400 eV was utilized in our simulations. The electronic structure was optimized until all forces were below 0.02 eV/Å. *Γ*-point AIMD simulations were performed with a 1 fs time step, and a Nosé thermostat was maintained at 300 K. The metal−water interfaces for Sn-based catalysts comprised a water layer of at least 10 Å in height, corresponding to a water density of 1 g/cm^3. Trasatti *et al.*[10] showed that U_{PZC} could be directly derived from the work function of a material in ion-free water ϕ using equation 2:

$$\phi = eU_{PZC} + e\phi_{SHE}, (2)$$

Here, ϕ_{SHE} represents the absolute potential energy of the SHE. It is important to note that the value of ϕ_{SHE} can vary depending on the experiment conducted (ranging from 4.3 to 4.8 eV). However, in this study, the recommended value of 4.44 eV by the International Union of Pure and Applied Chemistry (IUPAC) was used.

2. Supplementary tables and figures

Figure S1

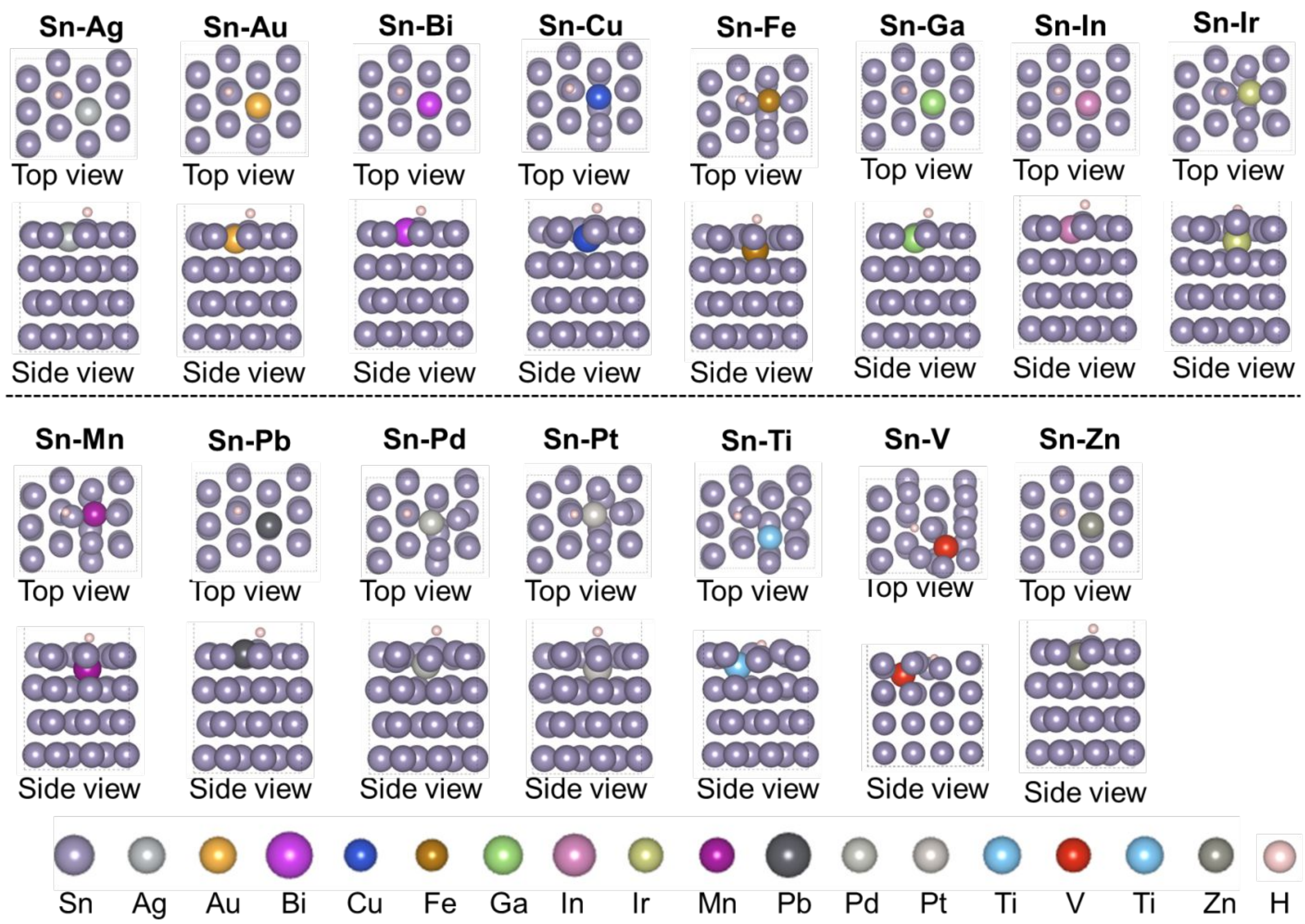

Figure S1. Optimized adsorption configuration of H at the Sn atomic site, including the top view and side view.

Figure S2

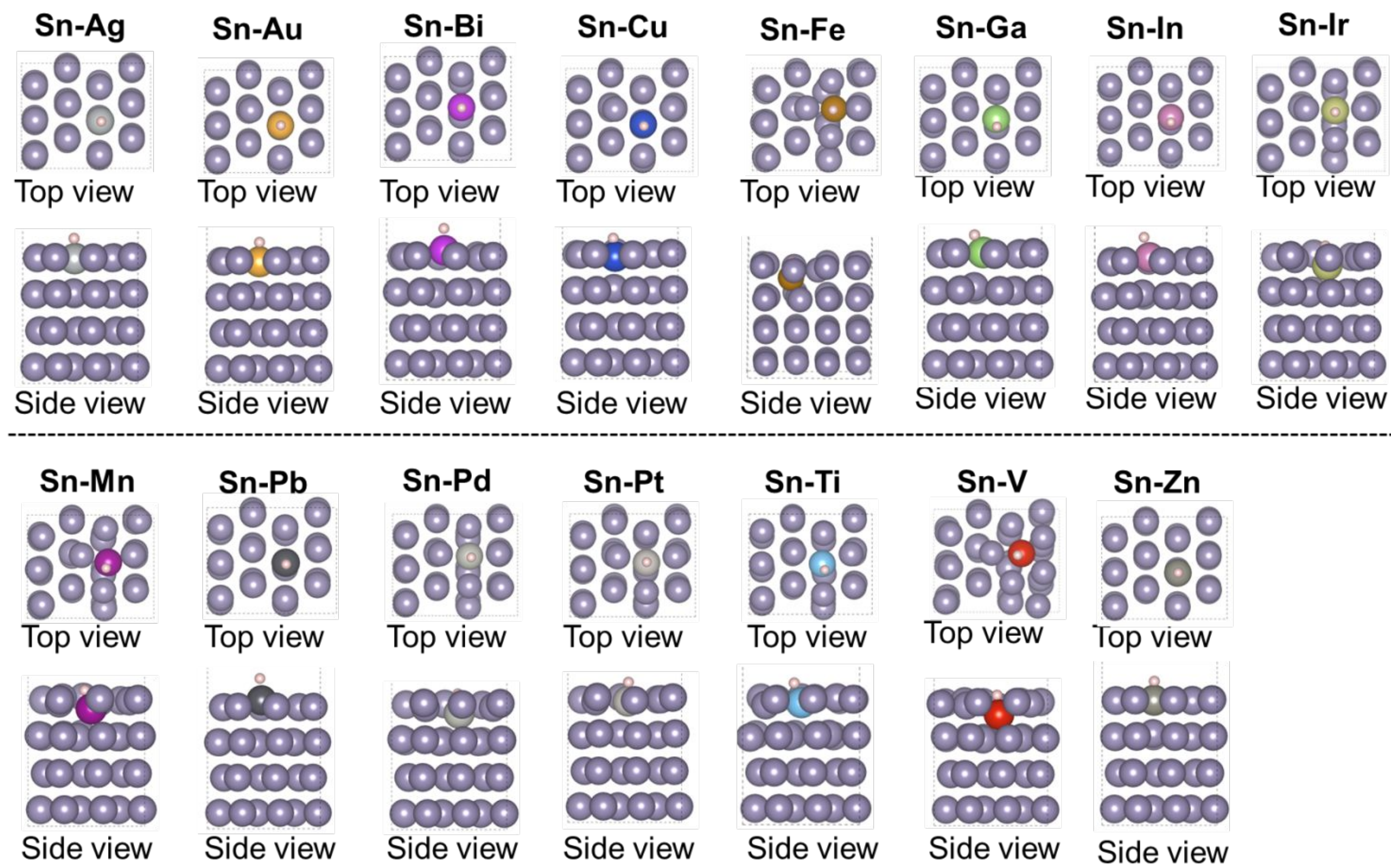

Figure S2. Optimized adsorption configuration of H at the dopant atomic site, including the top view and side view.

Figure S3

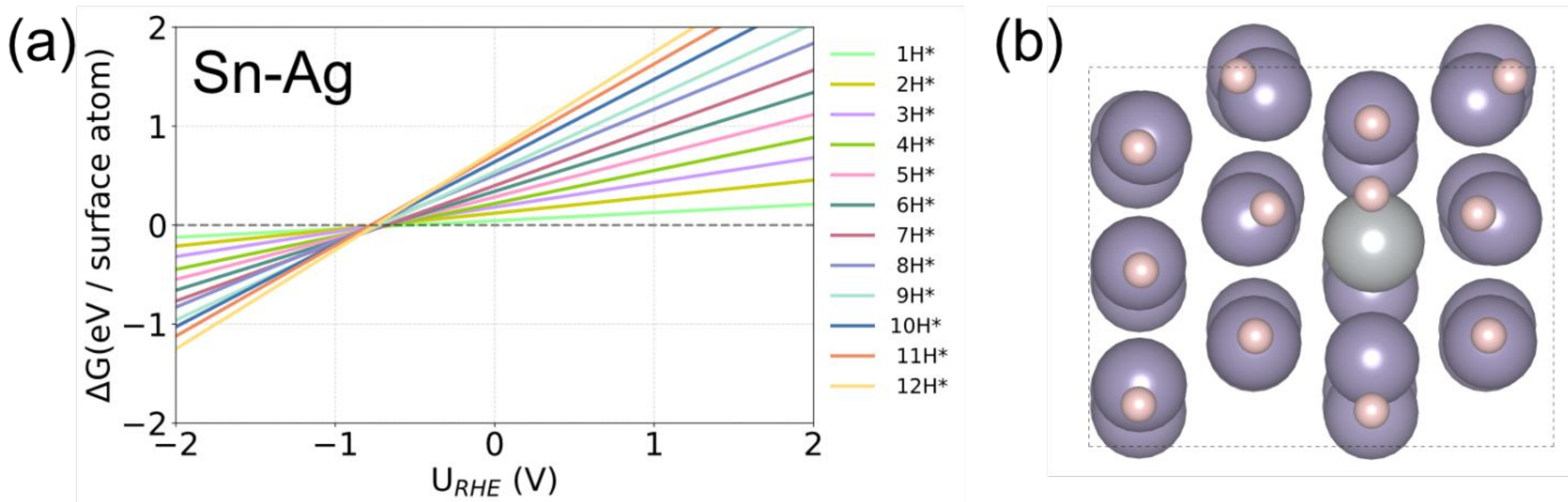

Figure S3. (a) One-dimensional Pourbaix diagram of the Ag-doped Sn(100) surface (denoted as Sn-Ag). (b) Top view of the most stable surface state at -1 V/RHE (pH = 0). Note: Sn sites are preferentially covered by H.

Figure S4

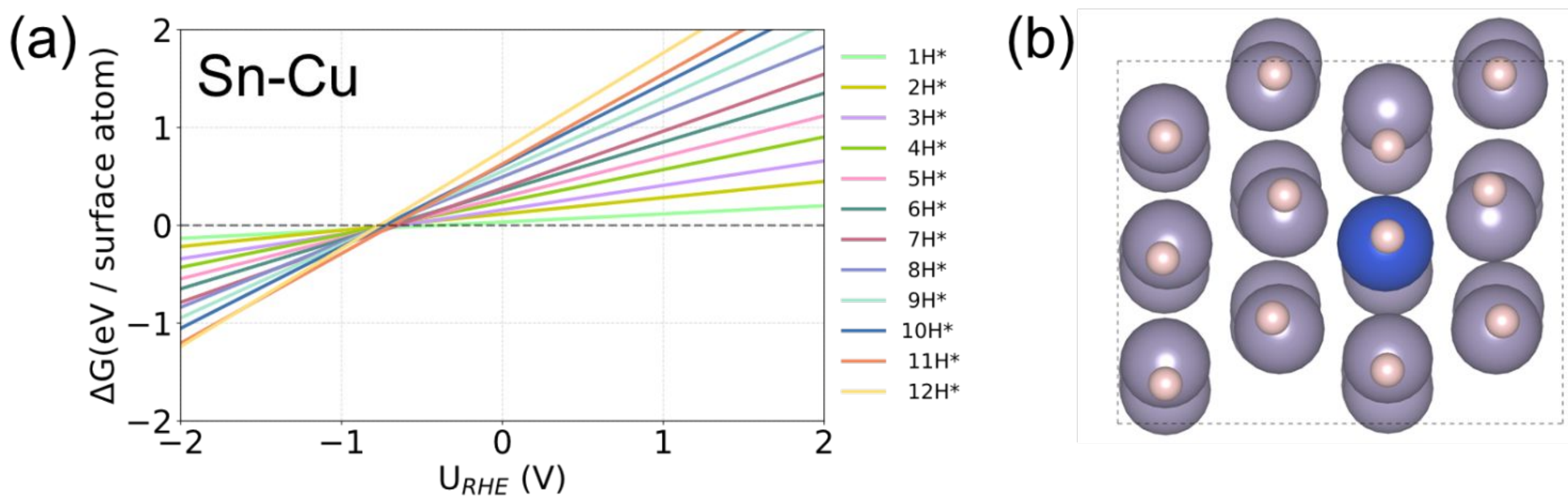

Figure S4. (a) One-dimensional Pourbaix diagram of the Cu-doped Sn(100) surface (denoted as Sn-Cu). (b) Top view of the most stable surface state at -1 V/RHE (pH = 0). Note: Sn sites are preferentially covered by H.

Figure S5

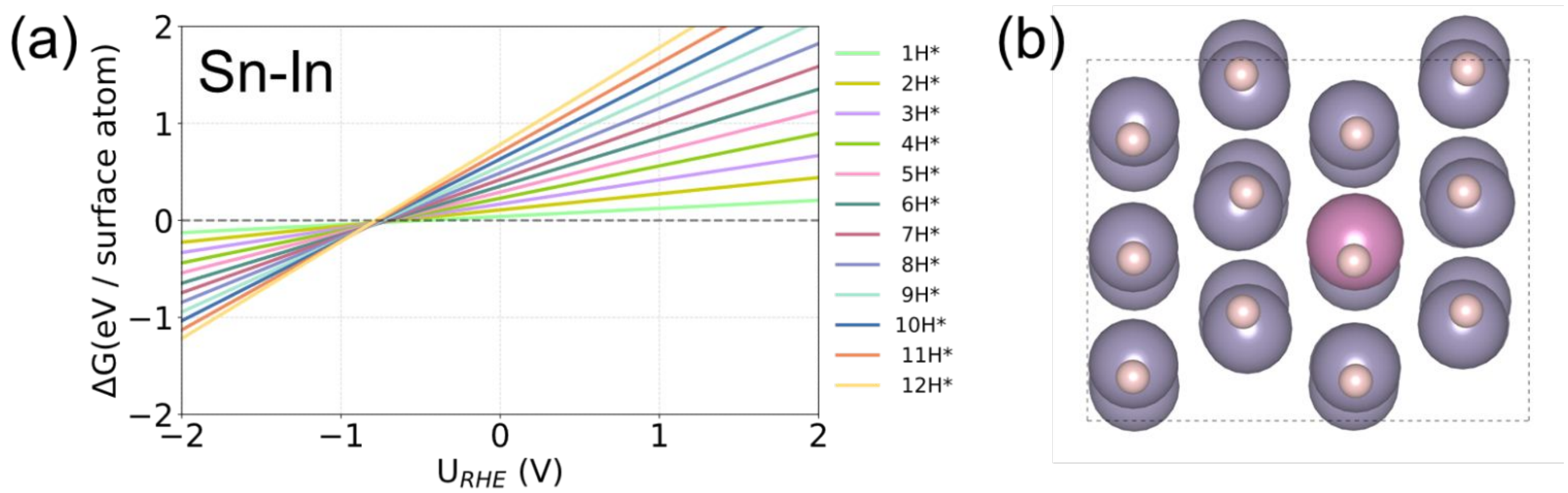

Figure S5. (a) One-dimensional Pourbaix diagram of the In-doped Sn(100) surface (denoted as Sn-In). (b) Top view of the most stable surface state at -1 V/RHE (pH = 0). Note: Sn sites are preferentially covered by H.

Figure S6

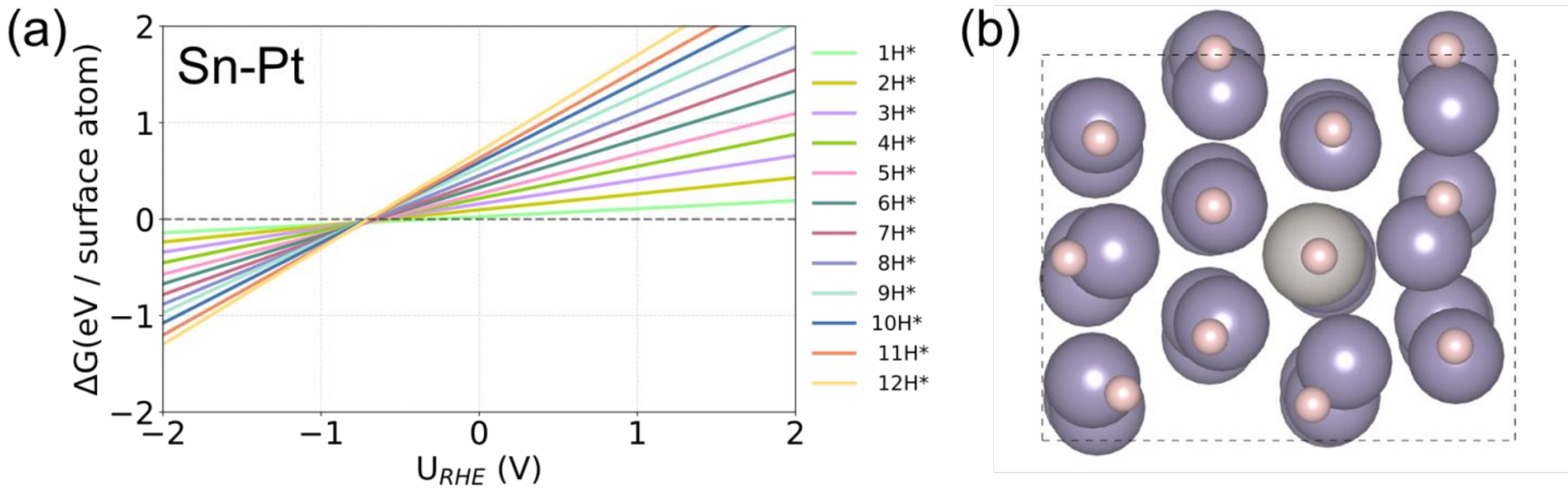

Figure S6. (a) One-dimensional Pourbaix diagram of the Pt-doped Sn(100) surface (denoted as Sn-Pt). (b) Top view of the most stable surface state at -1 V/RHE (pH = 0). Note: the dopant site is preferentially covered by H.

Figure S7

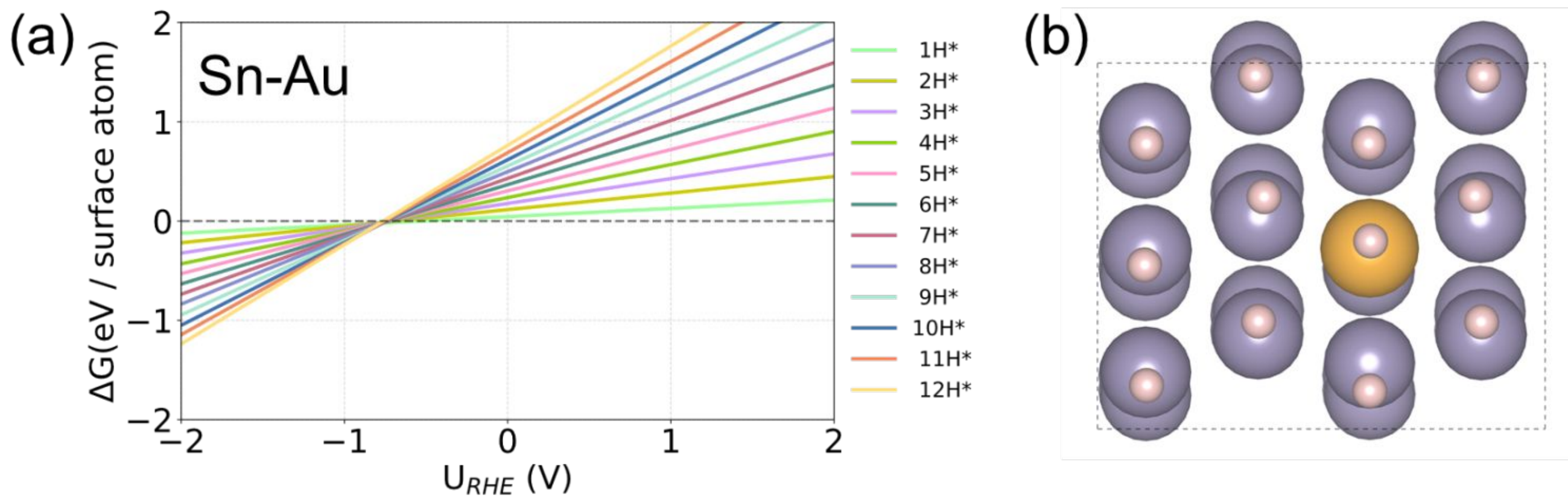

Figure S7. (a) One-dimensional Pourbaix diagram of the Au-doped Sn(100) surface (denoted as Sn-Au). (b) Top view of the most stable surface state at -1 V/RHE (pH = 0). Note: the dopant site is preferentially covered by H.

Figure S8

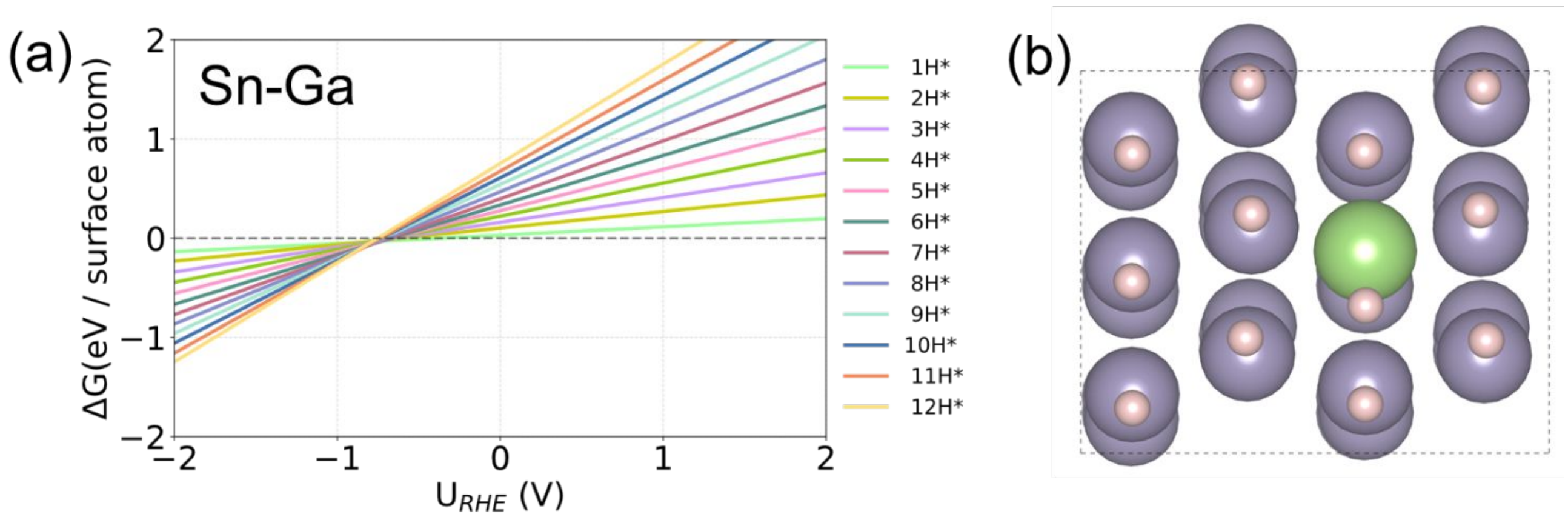

Figure S8. (a) One-dimensional Pourbaix diagram of the Ga-doped Sn(100) surface (denoted as Sn-Ga). (b) Top view of the most stable surface state at -1 V/RHE (pH = 0). Note: the dopant site is preferentially covered by H.

Figure S9

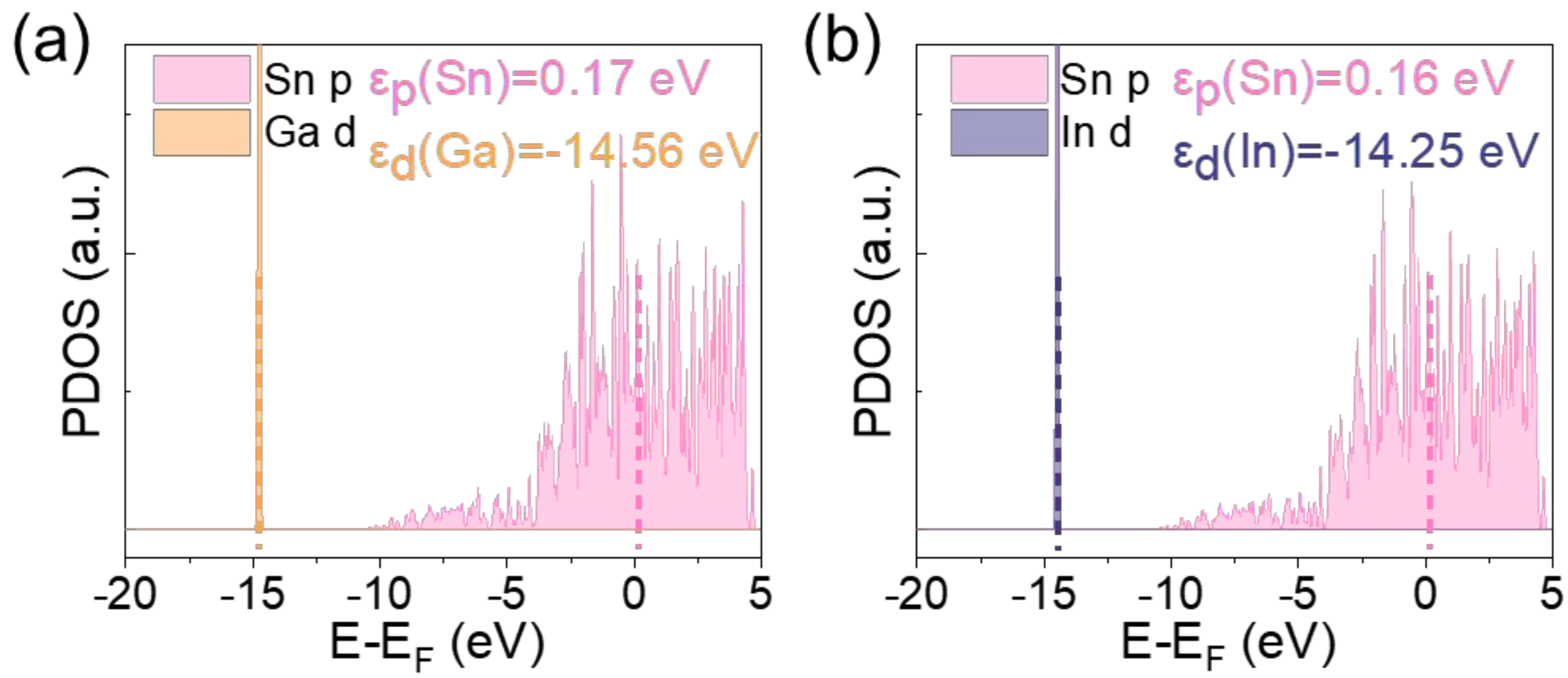

Figure S9. (a) PDOS of the Sn-Ga system and (b) PDOS of the Sn-In system. The dashed lines indicate the d-band centers.

Figure S10

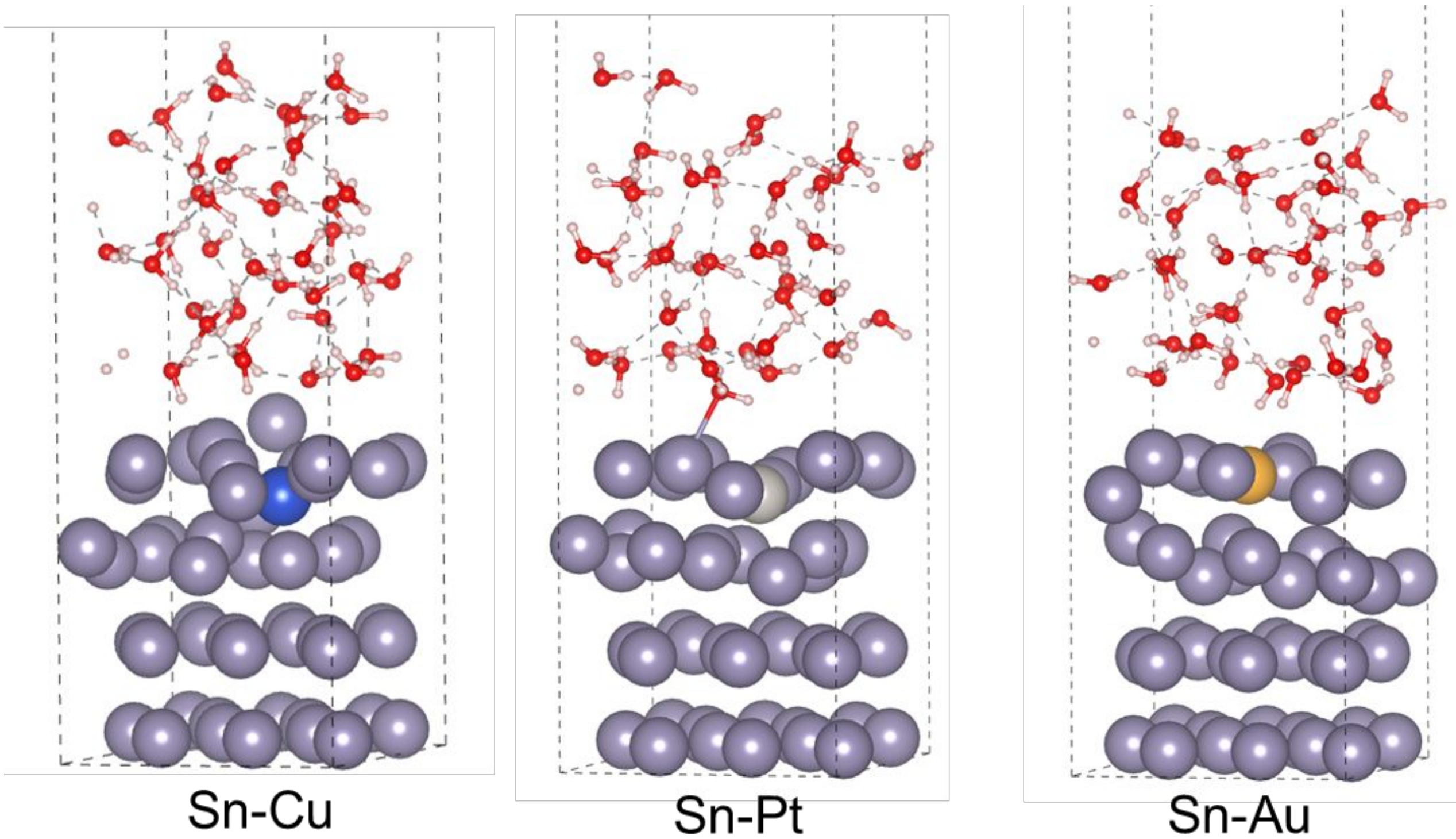

Figure S10. Schematic illustration of the water-metal interfacial structures of Sn-doped systems obtained from AIMD sampling, using Sn-Cu, Sn-Pt, and Sn-Au as representative examples.

Figure S11

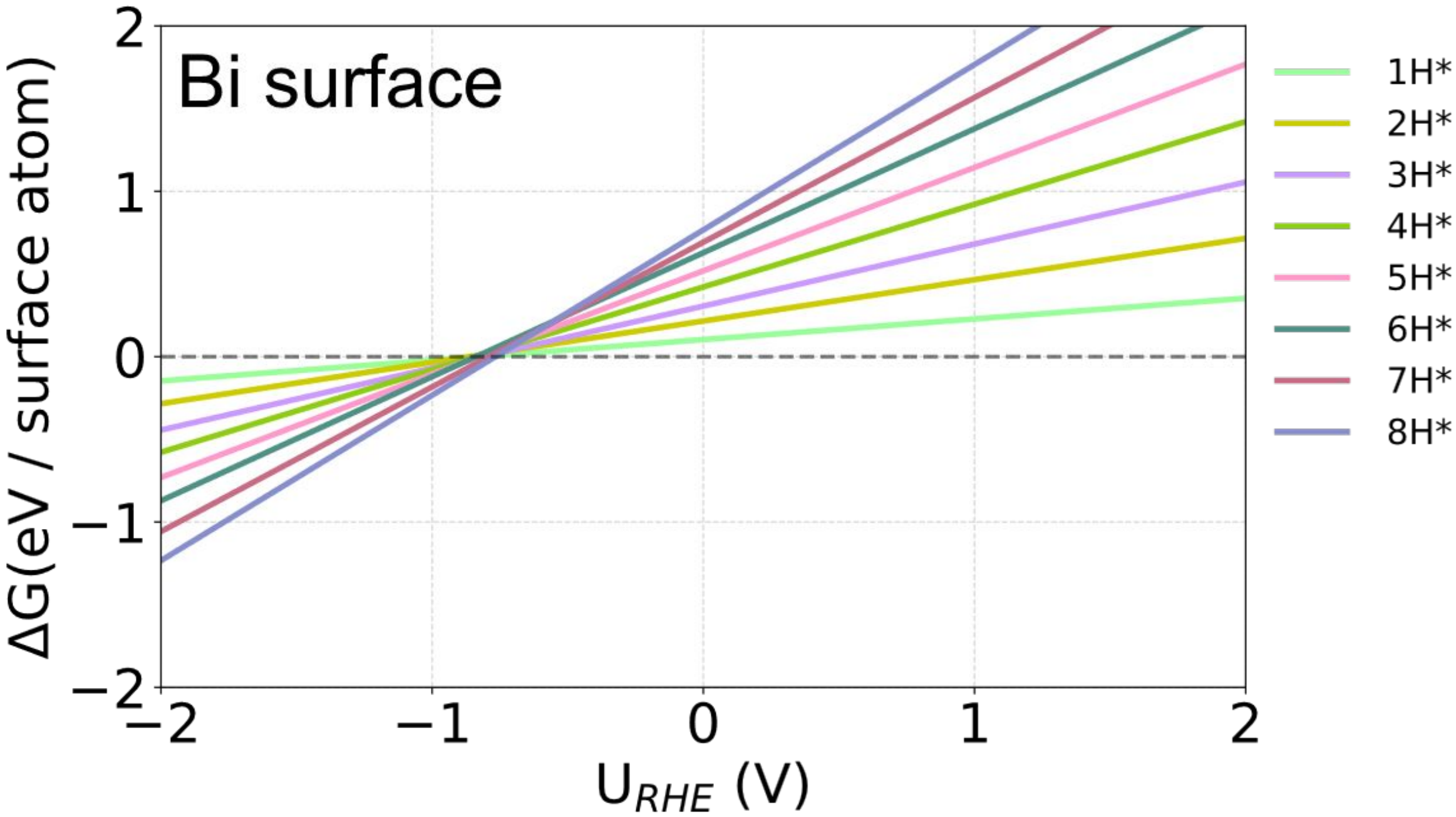

Figure S11. One-dimensional Pourbaix diagram of the Bi(012) surface.

Table S1

Table S1. Summary of the band centers in doped Sn(100) systems.

	**Sn**	**Dopant**		**H affinity**	
	p-band center	p-band center	d-band center	Sn site	Dopant site
Sn-Ag	0.20	0.46	-4.13	√	
Sn-Au	0.19	0.20	-3.72		√
Sn-Bi	0.11	-0.25	-22.39	√	
Sn-Cu	0.19	0.30	-2.37	√	
Sn-Fe	0.08	-1.41	-0.47		√
Sn-Ga	0.17	0.84	-14.56		√
Sn-In	0.16	0.75	-14.25	√	
Sn-Mn	0.14	-44.14	-0.10		√
Sn-Pb	0.23	0.64	-16.67	√	
Sn-Pd	0.07	-0.43	-2.35		√
Sn-Pt	0.07	-1.11	-2.77		√
Sn-Zn	0.18	0.81	-6.88	√	

Table S2

Table S2. Summary of the surface coverage statistics of H@Sn-doped systems across different potential ranges.

**Sn-Ag**		**Sn-Cu**		**Sn-In**	
Surface states	Applied potential (V/RHE)	Surface states	Applied potential (V/RHE)	Surface states	Applied potential (V/RHE)
Pristine	[-0.53, 2.0]	Pristine	[-0.40, 2.0]	Pristine	[-0.48, 2.0]
1 H*	[-0.70, -0.53]	1 H*	[-0.69, -0.40]	1 H*	[-0.75, -0.48]
4 H*	[-0.72, -0.70]	7 H*	[-0.74, -0.69]	6 H*	[-0.82, -0.75]
7 H*	[-0.84, -0.72]	11 H*	[-1.61, -0.74]	9 H*	[-0.89, -0.82]
12 H*	[-2.0, -0.84]	12 H*	[-2.0, -1.61]	11 H*	[-0.94, -0.89]
				12 H*	[-2.0, -0.94]

Table S3

Table S3. Summary of the surface coverage statistics of H@Dopant systems across different potential ranges.

**Sn-Pt**		**Sn-Au**		**Sn-Ga**	
Surface states	Applied potential (V/RHE)	Surface states	Applied potential (V/RHE)	Surface states	Applied potential (V/RHE)
Pristine	[-0.31, 2.0]	Pristine	[-0.53, 2.000]	Pristine	[-0.367, 2.0]
1 H*	[-0.71, -0.31]	1 H*	[-0.76, -0.53]	1 H*	[-0.73, -0.37]
5 H*	[-0.73, -0.71]	10 H*	[-0.85, -0.76]	6 H*	[-0.756, -0.73]
7 H*	[-0.74, -0.73]	11 H*	[-0.88, -0.85]	7 H*	[-0.84, -0.76]
11 H*	[-0.84, -0.74]	12 H*	[-2.0, -0.89]	11 H*	[-0.93, -0.84]
12 H*	[-2.0, -0.84]			12 H*	[-2.0, -0.93]

Table S4

Table S4. Calculated Gibbs free energies of *OCHO, *COOH, *H intermediates on doped Sn(100) and Bi(012) surface catalysts (in eV).

System	G_{*OCHO}	G_{*COOH}	G_{*H}
Sn-Ag	0.36	0.99	0.76
Sn-Bi	0.39	1.06	0.96
Sn-Cu	0.47	0.71	1.02
Sn-In	0.32	0.81	1.05
Sn-Mn	0.10	1.11	0.72
Sn-Pb	0.46	0.99	0.86
Sn-Zn	0.26	0.83	0.98
Sn-Au	0.22	0.85	0.46
Sn-Fe	0.04	0.37	2.32
Sn-Ga	0.31	0.39	0.98
Sn-Pd	0.40	0.54	0.98
Sn-Pt	-0.13	1.68	1.89
Bi-Ag	0.39	0.96	0.54
Bi-Au	0.28	0.89	0.36
Bi-Co	0.15	0.75	0.71
Bi-Cr	-0.03	0.68	1.13
Bi-Cu	0.39	0.92	0.87
Bi-Fe	0.23	0.97	0.70

Bi-Ga	0.50	0.92	0.46
Bi-In	0.51	0.92	0.45
Bi-Mn	0.32	0.98	0.82
Bi-Ni	0.45	1.00	0.66
Bi-Pd	0.50	0.98	0.65
Bi-Pt	0.24	0.76	0.31
Bi-Sn	0.56	1.08	0.57
Bi-Ti	-0.01	0.68	-0.02
Bi-V	0.26	0.76	0.93
Bi-Pb	0.11	0.80	0.89
Bi-Zn	0.16	0.90	0.58

Table S5

Table S5. Summary of the DFT-calculated energies, zero-point energies, heat capacities, and entropic contributions.[11, 12] Unit: eV.

**Species**	E^{DFT}	ZPE	$\int C_p dT$	$-TS$
CO_2 (g)	-22.51	0.31	0.10	-0.66
H_2 (g)	-7.04	0.28	0.09	-0.40
HCOOH(*l*)	-29.42	0.90	0.11	0.9
*OCHO	-	0.62	0.11	-0.24
*COOH	-	0.62	0.10	-0.19

References

(1) Le, J.; Iannuzzi, M.; Cuesta, A.; Cheng, J. Determining Potentials of Zero Charge of Metal Electrodes versus the Standard Hydrogen Electrode from Density-Functional-Theory-Based Molecular Dynamics. *Phys. Rev. Lett.* **2017**, *119* (1), 016801.

(2) Duan, S.; Xu, X.; Tian, Z.-Q.; Luo, Y. Hybrid Molecular Dynamics and First-Principles Study on the Work Function of a Pt(111) Electrode Immersed in Aqueous Solution at Room Temperature. *Phys. Rev. B* **2012**, *86* (4), 045450.

(3) Schnur, S.; Groß, A. Properties of Metal–Water Interfaces Studied from First Principles. *New J. Phys.* **2009**, *11* (12), 125003.

(4) Sakong, S.; Groß, A. The Electric Double Layer at Metal-Water Interfaces Revisited Based on a Charge Polarization Scheme. *J. Chem. Phys.* **2018**, *149* (8), 084705.

(5) Hammer, B.; Hansen, L. B.; Nørskov, J. K. Improved Adsorption Energetics within Density-Functional Theory Using Revised Perdew-Burke-Ernzerhof Functionals. *Phys. Rev. B* **1999**, *59* (11), 7413.

(6) Perdew, J. P.; Burke, K.; Ernzerhof, M. Generalized Gradient Approximation Made Simple. *Phys. Rev. Lett.* **1996**, *77* (18), 3865.

(7) Grimme, S.; Antony, J.; Ehrlich, S.; Krieg, H. A Consistent and Accurate Ab Initio Parametrization of Density Functional Dispersion Correction (DFT-D) for the 94 Elements H-Pu. *J. Chem. Phys.* **2010**, *132* (15), 154104.

(8) Heenen, H. H.; Gauthier, J. A.; Kristoffersen, H. H.; Ludwig, T.; Chan, K. Solvation at Metal/Water Interfaces: An Ab Initio Molecular Dynamics Benchmark of Common Computational Approaches. *J. Chem. Phys.* **2020**, *152* (14), 144703.

(9) Sakong, S.; Forster-Tonigold, K.; Groß, A. The Structure of Water at a Pt(111) Electrode and the Potential of Zero Charge Studied from First Principles. *J. Chem. Phys.* **2016**, *144* (19), 194701.

(10) Trasatti, S. Structure of the Metal/Electrolyte Solution Interface: New Data for Theory. *Electrochim. Acta* **1991**, *36* (11), 1659–1667.

(11) Christensen, R.; Hansen, H. A.; Vegge, T. Identifying Systematic DFT Errors in Catalytic Reactions. *Catal. Sci. Technol.* **2015**, *5* (11), 4946–4949.

(12) Chan, K.; Tsai, C.; Hansen, H. A.; Nørskov, J. K. Molybdenum Sulfides and Selenides as Possible Electrocatalysts for CO_2 Reduction. *ChemCatChem* **2014**, *6* (7), 1899–1905.
